# Assessment of integrated patterns of human-animal-environment health: a holistic and stratified analysis

**DOI:** 10.1186/s40249-023-01069-0

**Published:** 2023-03-14

**Authors:** Zhao-Yu Guo, Jia-Xin Feng, Lin Ai, Jing-Bo Xue, Jing-Shu Liu, Xiao-Xi Zhang, Chun-Li Cao, Jing Xu, Shang Xia, Xiao-Nong Zhou, Jin Chen, Shi-Zhu Li

**Affiliations:** 1 National Institute of Parasitic Diseases, Chinese Centre for Disease Control and Prevention (Chinese Centre for Tropical Diseases Research), NHC Key Laboratory of Parasite and Vector Biology, WHO Collaborating Centre for Tropical Diseases, National Centre for International Research On Tropical Diseases, Shanghai, 200025 China; 2 School of Global Health, Chinese Centre for Tropical Diseases Research, Shanghai Jiao Tong University School of Medicine, Shanghai, 200025 China

**Keywords:** One Health, Evaluation framework, Economic disparity, Global One Health index

## Abstract

**Background:**

Data-driven research is a very important component of One Health. As the core part of the global One Health index (GOHI), the global One Health Intrinsic Drivers index (IDI) is a framework for evaluating the baseline conditions of human-animal-environment health. This study aims to assess the global performance in terms of GOH-IDI, compare it across different World Bank regions, and analyze the relationships between GOH-IDI and national economic levels.

**Methods:**

The raw data among 146 countries were collected from authoritative databases and official reports in November 2021. Descriptive statistical analysis, data visualization and manipulation, Shapiro normality test and ridge maps were used to evaluate and identify the spatial and classificatory distribution of GOH-IDI. This paper uses the World Bank regional classification and the World Bank income groups to analyse the relationship between GOH-IDI and regional economic levels, and completes the case studies of representative countries.

**Results:**

The performance of One Health Intrinsic Driver in 146 countries was evaluated. The mean (standard deviation, *SD*) score of GOH-IDI is 54.05 (4.95). The values (mean *SD*) of different regions are North America (60.44, 2.36), Europe and Central Asia (57.73, 3.29), Middle East and North Africa (57.02, 2.56), East Asia and Pacific (53.87, 5.22), Latin America and the Caribbean (53.75, 2.20), South Asia (52.45, 2.61) and sub-Saharan Africa (48.27, 2.48). Gross national income per capita was moderately correlated with GOH-IDI (*R*^*2*^ = *0.651,* Deviance explained = *66.6%, P* < 0.005). Low income countries have the best performance in some secondary indicators, including Non-communicable Diseases and Mental Health and Health risks. Five indicators are not statistically different at each economic level, including Animal Epidemic Disease, Animal Biodiversity, Air Quality and Climate Change, Land Resources and Environmental Biodiversity.

**Conclusions:**

The GOH-IDI is a crucial tool to evaluate the situation of One Health. There are inter-regional differences in GOH-IDI significantly at the worldwide level. The best performing region for GOH-IDI was North America and the worst was sub-Saharan Africa. There is a positive correlation between the GOH-IDI and country economic status, with high-income countries performing well in most indicators. GOH-IDI facilitates researchers' understanding of the multidimensional situation in each country and invests more attention in scientific questions that need to be addressed urgently.

**Graphical Abstract:**

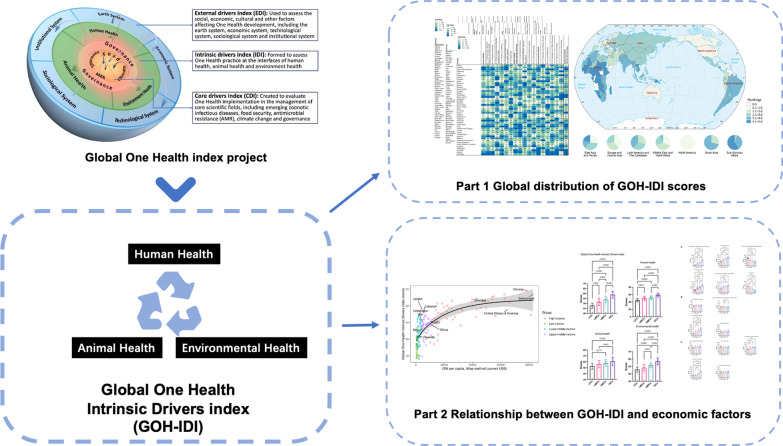

**Supplementary Information:**

The online version contains supplementary material available at 10.1186/s40249-023-01069-0.

## Background

The global pandemic of coronavirus disease 2019 (COVID-19) has increased the awareness of the links and interactions between human health, animal health, and environmental health. To address complex health issues, many experts and academics have proposed the concept of "One Health". This approach considers the interconnectedness of human, animal, and environmental health and seeks to improve overall well-being without compromising any of these elements. By studying and managing health in this holistic way, the effectiveness of interventions is enhanced [1, 2].

Many countries and research institutions have different definitions of health and sustainability, deriving many indicator systems related to One Health but fundamentally different. There are currently a variety of datasets related to One Health, including Sustainable Development Goals (SDGs), Gross Domestic Product (GDP), Human Development Index (HDI), Environmental Performance Index (EPI) and Global Burden of Disease [3–9]. In addition, there are a number of exploratory studies of the One Health concept [10–16]; some of them have been carried out as field surveys [12, 13]. A global assessment is important, which could build a large network of mutual trust and promote the spread of the One Health concept. In 2022, a new global One Health assessment framework was developed [17, 18]. Global One Health index (GOHI) consists of three parts: core drivers index, intrinsic drivers index, and external drivers index [17]. However, the antecedent study describes the data holistically and does not discuss the intrinsic drivers index in detail [17]. The Intrinsic drivers index assesses the actual situation at this stage across regions and countries, making society aware of the pressing socio-economic issues of the day and promoting the implementation and development of the One Health action. Therefore, we conducted this study and accomplished data stratification and analyze using the socioeconomic factor Gini coefficient.

Global One Health Intrinsic Drivers index (GOH-IDI) focuses more on the interface of human health, animal health and ecosystem diversity, and environmental health [19]. "Intrinsic" represents the outcome indicator in the GOHI. Traditional indicator systems generally contain three components, including structure (stable components of the system), process (interventions on the system), and outcome (impact of the indicator on the system) [20, 21]. A separate assessment of outcome indicators and intervention indicators is more conducive to researchers and policymakers to understand the implications of the data [1, 8, 22, 23].

In earlier studies, our team developed the framework and weights of GOH-IDI, which accomplished by the same key members used grounded theory (GT) method, fuzzy analytical hierarchy process (FAHP) and entropy weight method (EWM) to construct the indicators and calculate the weights of GOH-IDI [19]. This study described Global distribution of GOH-IDI, analyzed correlation between GOH-IDI score and Gini coefficient and made policy recommendations in the perspective of global realities and typical case discussions.

## Methods

### Data collection and resources

The raw data collection and calculation was constructed in five steps, including framework formulation, indicator selection, database building, weight determination and GOHI scores calculation [17, 18]. The data related to the GOH-IDI project is stored in GitHub (https://github.com/DayuGuo/G2-IDI). GOH-IDI's database consists of 13 open sources and reliable databases (Table 1) [17, 18].

**Table 1:** Main database

Dimension	Database	Source
Human Health	SDGs Dashboard	https://dashboards.sdgindex.org/
	WHO	https://www.who.int/data
	IHME-GBD	https://www.healthdata.org/gbd/2019
Animal Health and Ecosystem Diversity	EMPRES-I	https://empres-i.apps.fao.org/
	OIE-WAHIS	https://wahis.woah.org/#/home
	Environmental Performance Index	https://epi.yale.edu/
	Our World in Data	https://ourworldindata.org/
Environmental Health	World Bank	https://data.worldbank.org/indicator
	State of Global Air	https://www.stateofglobalair.org/
	Global Climate Risk Index	https://www.germanwatch.org/en/cri
	Environmental Performance Index	https://epi.yale.edu/
	Our World in Data	https://ourworldindata.org/
	SDGs Dashboard	https://dashboards.sdgindex.org/

*EMPRES-I* Emergency Prevention Programme for Transboundary Animal Diseases, *OIE-WAHIS* OIE World Animal Health Information System

### GOH-IDI framework overview

Our team have established a scientific standard to evaluate the intrinsic drivers and a scientific standard to measure the development level in different regions for One Health [19]. The specific procedure for the construction and score calculation of the GOH-IDI framework has been published and is organized in Additional file 1 and Additional file 2 [17, 18]. The indictor scheme for GOH-IDI composes of three first-level indicators, 15 second-level indicators, 61 third-level indicators. Additional file 1 contains the detailed indicators and weights of GOH-IDI [19].

### Visualization analysis

Version control of all data in this project is hosted on GitHub Desktop 2.9.11 (GitHub Incorporated, USA). Data and algorithms are open sources (https://github.com/DayuGuo/OHI-IDI-Animal-Environmental). Data were analyzed using R studio 2021.09.1 (Posit Software, Boston, USA), R version 4.1.2 (Lucent Technologies, Jasmine Mountain, USA). R Packages used in the analyze include tidyverse, ggrepel, ggplot2, coplot, ggstatsplot, palmerpenguins, readr, and esquisse. Methods of data analyze, and evaluation include descriptive statistics, data visualizations and data shaping. Heat maps are used to assess differences between indicators at each level. To observe the differences between regions and countries across the globe, we mapped the spatial distribution using ArcGIS 10.5 (ESRI, Redlands, USA). The Shapiro normality test is used to test whether the data is normally distributed. GOH-IDI data used in this paper are normally distributed, so means and standard deviations (*SD*) are used, and GraphPad Prism 9.3.1 (Graphpad Software Incorporated, USA) is used for those graphs. To analyze the data at a hierarchical level, we have used data from the World Bank database (https://data.worldbank.org/indicator). Gross national income (GNI) per capita (Atlas method) is used because it is an internationally accepted and comparable indicator. Generalized Additive Models (GAM) were used to analyze the relationship between GOH-IDI and GNI per capita. The country with the highest score in each World Bank income level was selected for case studies. The data analyze of the case studies was done through Excel Version 2107 (Microsoft Windows, USA). Countries in different geographical regions have different economic profiles. Its economic level determines a country's resources dedicated to governance. Analyzing the current situation of each country in conjunction with the economic level and GOH-IDI performance can lead to more effective strategies. The World Bank assigns the world's economies to four income groups based on GNI per capita, including high income countries (HICs), upper middle income countries (UMICs), lower middle income countries (LMICs) and low income countries (LICs) (see Additional file 2 for details).

## Results

### Global distribution of GOH-IDI scores

**Fig. 1:**
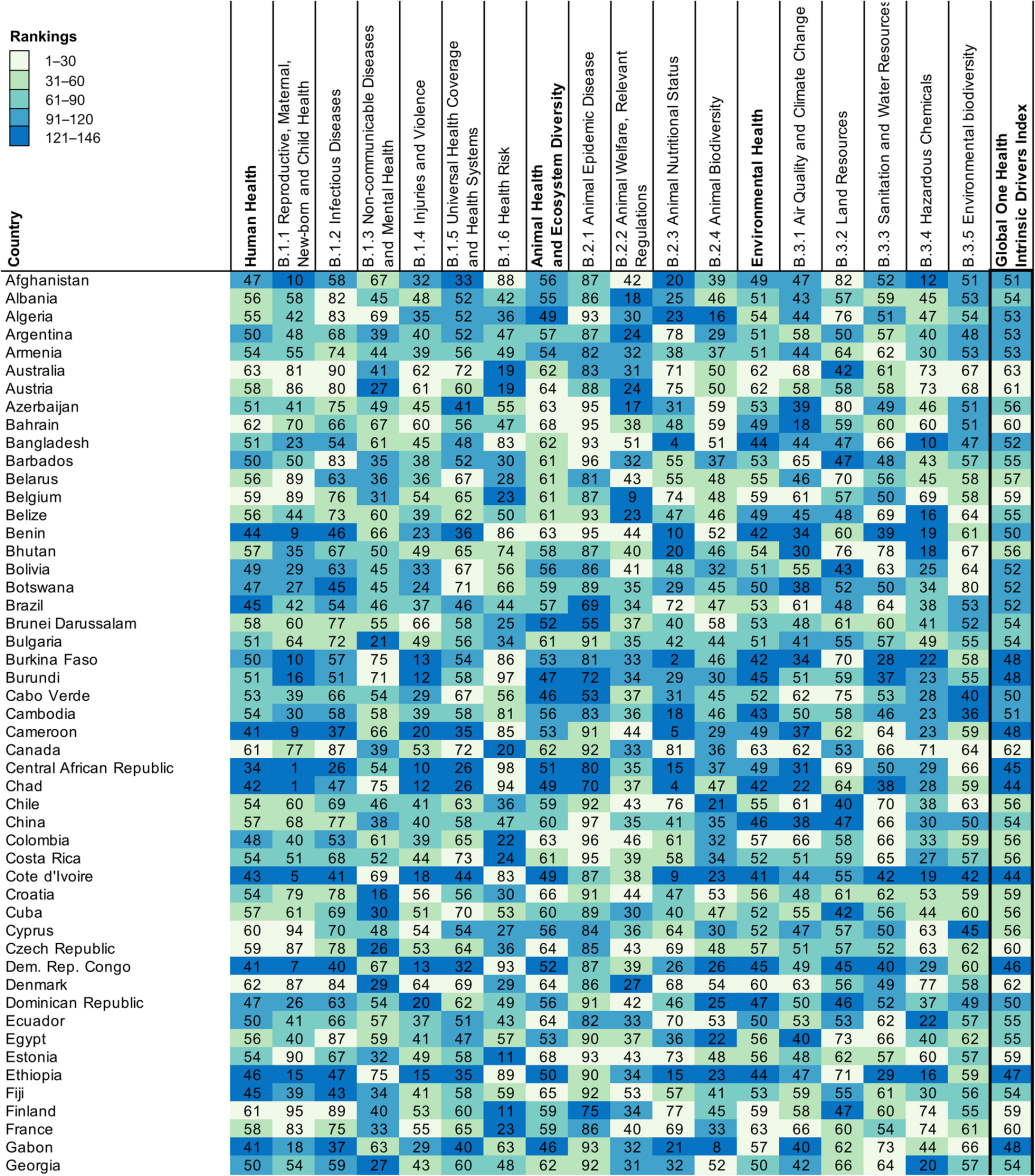

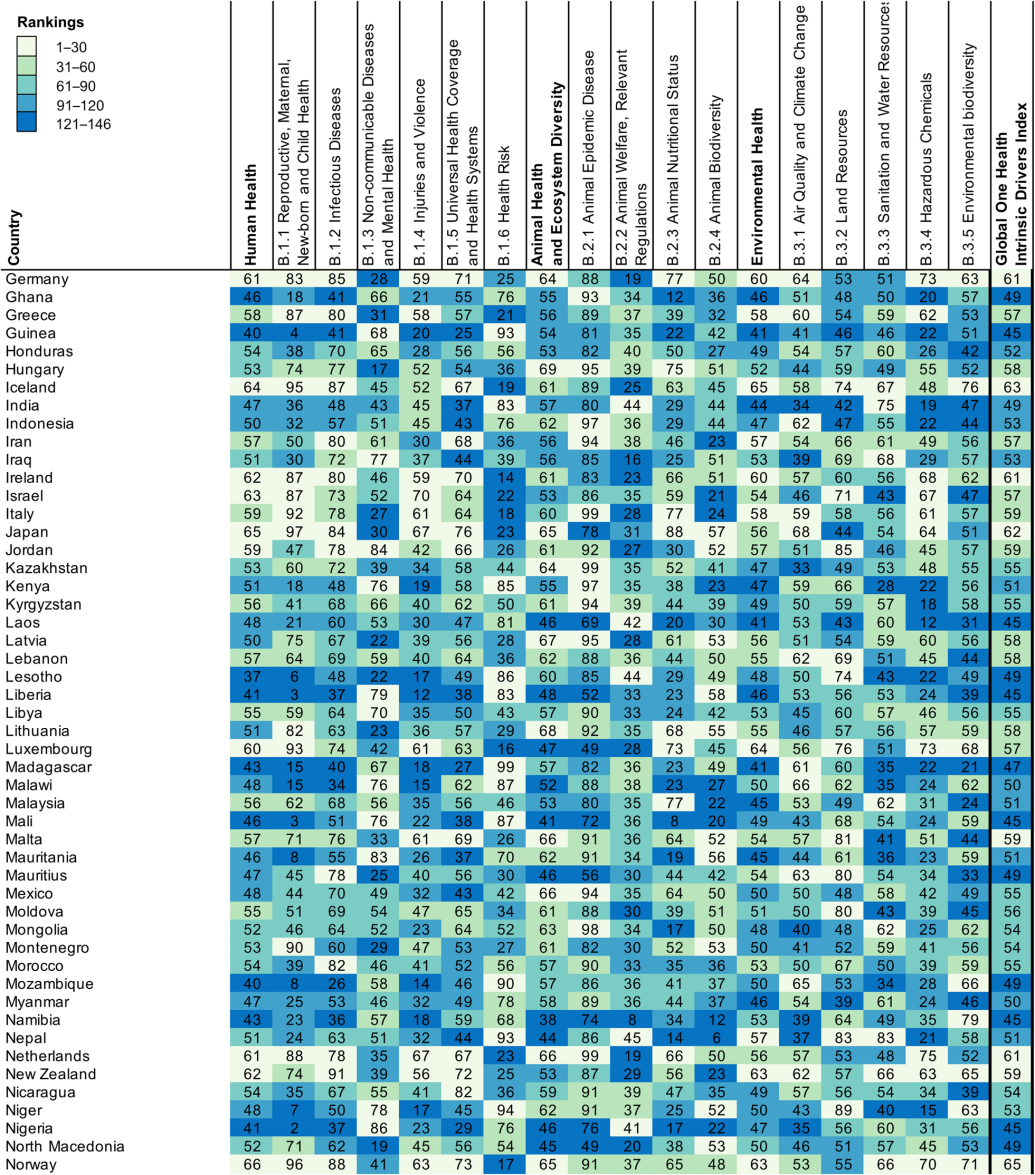

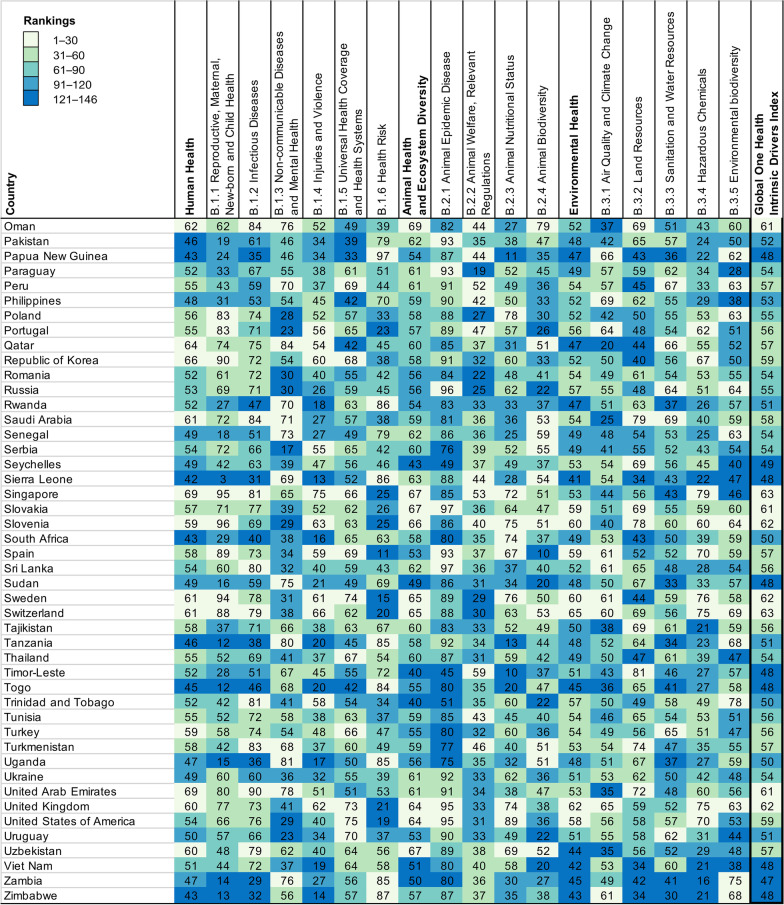
Performance on GOH-IDI. Countries are listed in alphabetical order, and are divided into five ranking groups. The numbers in Fig. 1 represent the scores of each country, and the depth of color represents the ranking of the country for that score. The raw data information contained in each indicator is shown in the Additional files 1, 2, 3. *GOH-IDI* Global One Health Intrinsic Driver Index

A total of 146 countries worldwide were included in this study, of which 19 were in East Asia and Pacific, 47 were in Europe and Central Asia, 18 were in Latin America and the Caribbean, 16 were in Middle East and North Africa, 16 were in Middle East and North Africa, 2 were in North America, 7 were in South Asia, and 37 were in sub-Saharan Africa (see Additional file 2 for details). The mean (*SD*) score of GOH-IDI is 54.05 (4.95), the lowest score is 44.11 and the highest score is 64.51 (Fig. 1). The mean (*SD*) score of Human Health is 52.73 (6.79). The mean (*SD*) score of Animal Health and Ecosystem Diversity is 57.68 (6.54). The mean (*SD*) score of Environmental Health is 51.74 (5.61).

**Fig. 2:**
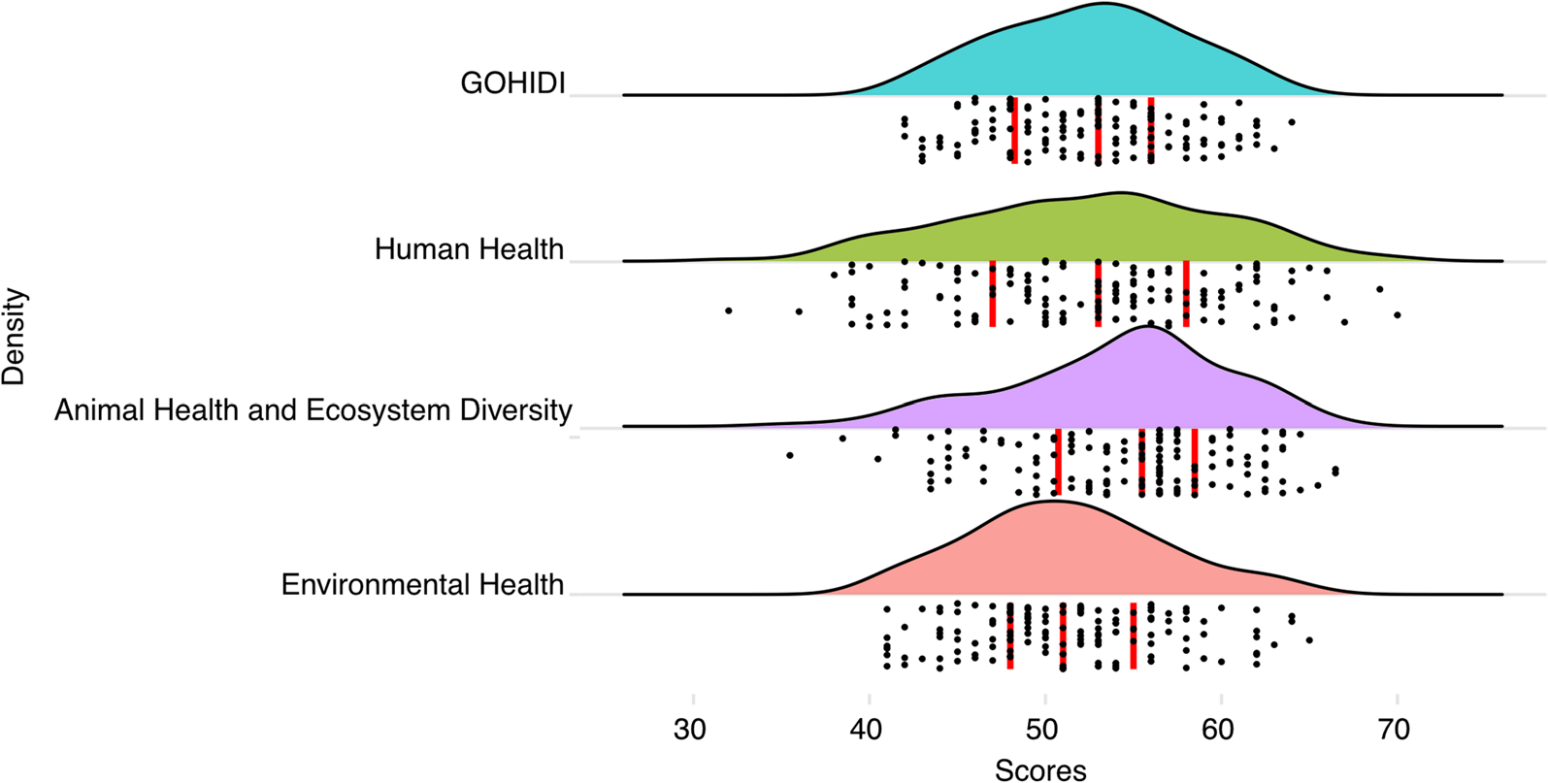
Dimensional Distribution of GOH-IDI. Three red lines in each ridge map corresponds to the first, second, and third quartile. The black dots represent the distribution of scores, with more dots indicating more countries with that score. GOH-IDI scores used in this paper are normally distributed. *GOH-IDI* Global One Health Intrinsic Driver Index

The Shapiro–Wilk normality test proved that the GOH-IDI score profile fits a normal distribution (W = 0.981, *P* = 0.047), as do human health and environmental health. The animal health section has many qualitative indicators unsuitable for normal distribution analyze (Fig. 2).

**Fig. 3:**
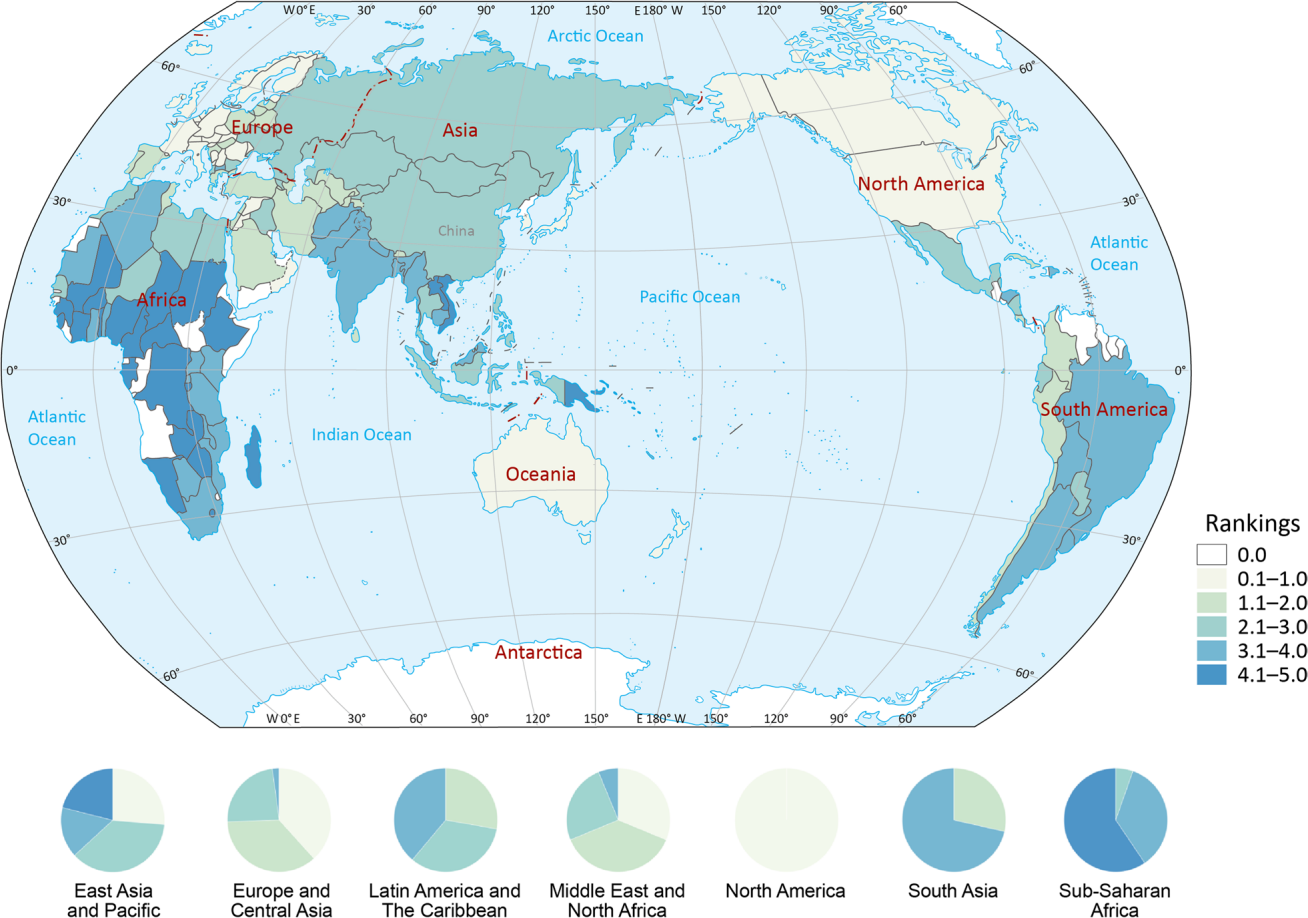
Regional rankings map of GOH-IDI. Heat map (Fig. 1) and spatial distribution (Fig. 3) were divided into five groups by score ranking with the same criteria. The numbers in Fig. 1 represent the scores of each country, and the depth of color represents the ranking of the country for that score. The pie charts represent the distribution of GOH-IHI scores within each region. The raw data information contained in each indicator is shown in the Additional files 1, 2, 3. *GOH-IDI* Global One Health Intrinsic Driver Index

Global distribution of scores of GOH-IDI is illustrated in Fig. 3. This study includes two countries in North America, the scores of these two countries are ranked between 1 and 30. Most of the sub-Saharan Africa (SSA) countries (59.5%) rank between 121 and 146. The highest-scoring country in SSA is Senegal (53.56), and the lowest score is Cote d'Ivoire (44.11). The highest-scoring country in North America is Canada (62.10), and the lowest is the United States of America (58.77). The highest-scoring country in South Asia is Bhutan (56.09), and the lowest is India (49.36). The highest-scoring country in the Middle East and North Africa is the United Arab Emirates (61.15), and the lowest is Algeria (52.74). In Latin America and the Caribbean, the country with the highest score is Peru (56.64), and the lowest score is Trinidad and Tobago (49.56). The highest-scoring country in East Asia and Pacific is Singapore (62.88), and the lowest is Laos (45.10). Among Europe and Central Asia, the highest-scoring country is Norway (64.51), and the lowest is North Macedonia (49.17) (Fig. 3).

**Fig. 4:**
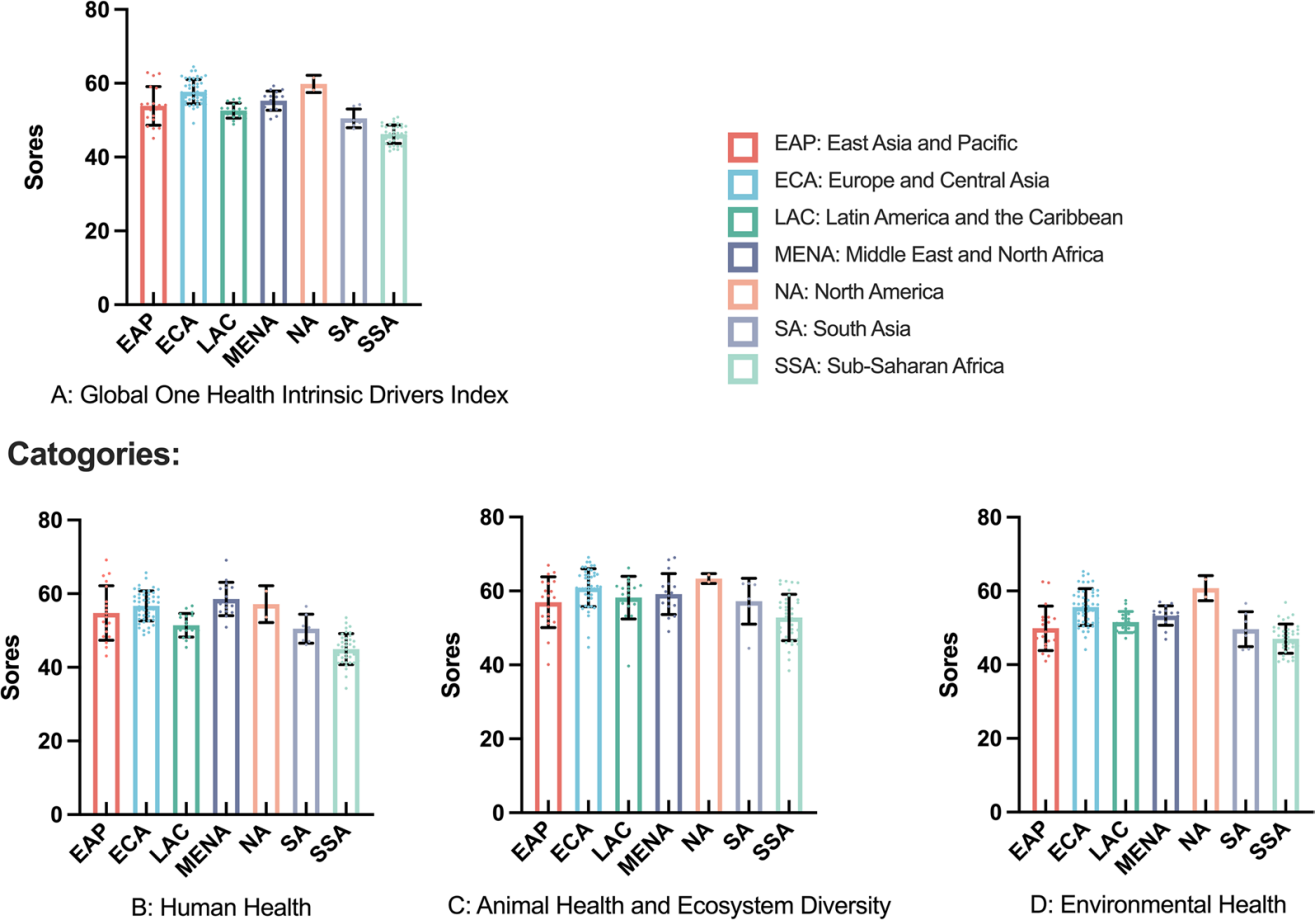
Mean (*SD*) of GOH-IDI and Categories scores between regions. Note: The different bar chart colors represent different regions. The error line represents the standard deviation. The dots represent the distribution of the data and correspond to the vertical coordinates. Detailed data are listed in Additional file 2. Descriptive analyze are listed in Additional file 3. *GOH-IDI* Global One Health Intrinsic Driver Index, *SD* standard deviation

In the World Bank regional classification, the mean (*SD*) values of GOH-IDI scores by region are (high to low): North America is 60.44 (2.36); Europe and Central Asia is 57.73 (3.29); Middle East and North Africa is 57.02 (2.56); East Asia and Pacific is 53.87 (5.22); Latin America and the Caribbean is 53.75 (2.20); South Asia is 52.45 (2.61); SSA is 48.27 (2.48). SSA mean scores are statistically different from all six other regions, and more significant in the Human Health section (*P* < 0.05). The mean (*SD*) of SSA in Human Health is 44.94 (4.20) (Fig. 4).

### Relationship between GOH-IDI scores and economic factors

**Fig. 5:**
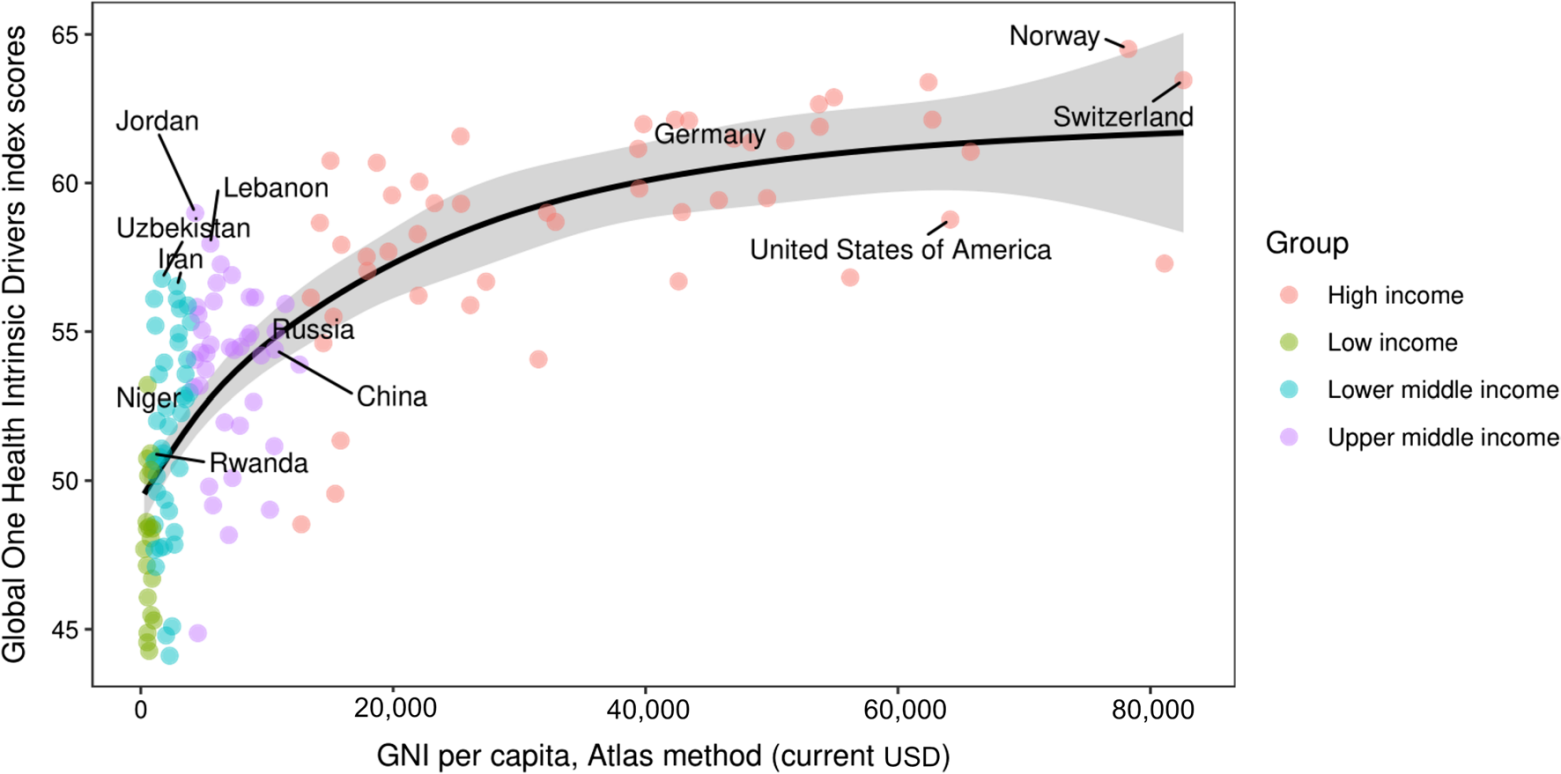
Correlation analyze of GNI per capita and GOH-IDI scores. The World Bank income group is distinguished by four colors (see Additional file 2 for details). Correlation analyze GOH-IDI scores and GNI per capita, Atlas method (current USD). *GNI* Gross national income, *GOH-IDI* Global One Health Intrinsic Driver Index

In the correlation analyze section, GNI per capita was moderately correlated with GOH-IDI by GAM, *R*^2^ = 0.651, Deviance explained = 66.6%, *P* < 0.005 (Fig. 5).

**Fig. 6:**
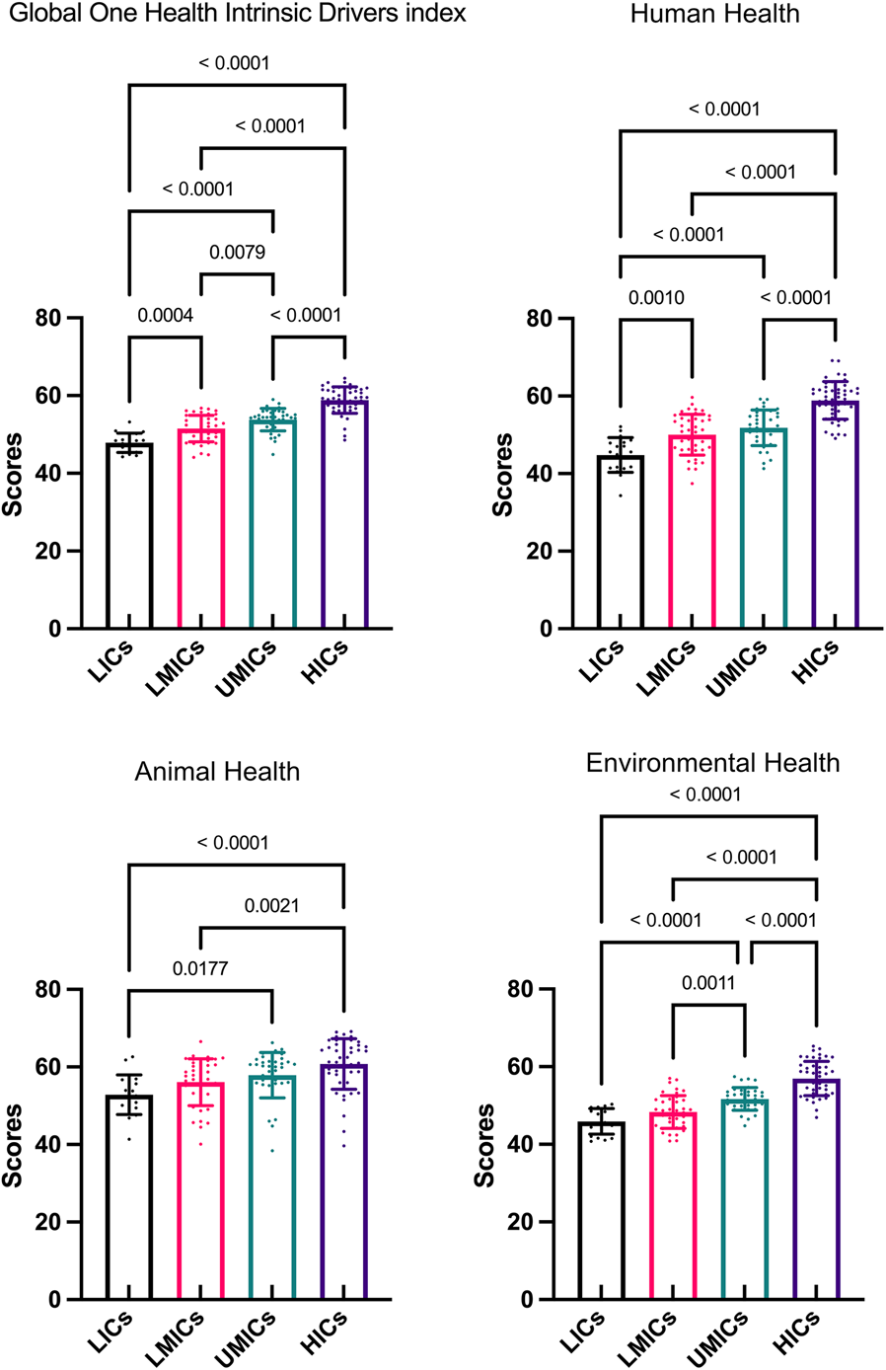
Mean (*SD*) of GOH-IDI and Categories scores in different World Bank income groups. The different colors of the bar chart represent different regions. The error line represents the standard deviation. The dots represent the distribution of the data and correspond to the vertical. Detailed data are listed in Additional file 2. Descriptive analyze are listed in Additional file 3. *SD* Standard deviation, *GOH-IDI* Global One Health Intrinsic Driver Index, *LICs* Low-income countries, *LMICs* Lower middle-income countries, *UMICs* Upper middle-income countries, *HICs* High income-countries

A total of 146 countries worldwide were included in this study, of which 19 were LICs, 41 were LMICs, 38 were UMICs, 48 were HICs. The mean (*SD*) values of GOH-IDI scores by World Bank income groups are (high to low): HICs are 58.86 (3.42); UMICs are 53.82 (2.90); LMICs are 51.49 (3.42); LICs are 47.86 (2.46). All four data sets were statistically different (Fig. 6). The LICs-HICs and LMICs-HICs were statistically significant differences in the Human Health, Animal Health and Environmental Health score profiles (Fig. 6) (*P* < 0.01). In GOH-IDI, the highest country of each different World Bank group is Norway (64.51), Jordan (58.99), Uzbekistan (56.78) and Niger (53.21). In GOH-IDI-Human Health, the highest country among each different World Bank group is Singapore (69.17), Jordan (59.20), Uzbekistan (59.68) and Rwanda (52.09). In GOH-IDI-Animal Health, the highest country of each different World Bank group is Hungary (69.12), Mexico (66.25), Uzbekistan (66.53) and Sierra Leone (62.62). In GOH-IDI-Environmental Health, the highest country of each different World Bank group is Switzerland (65.28), Colombia (57.42), Iran (57.03) and Malawi (50.37).

**Fig. 7:**
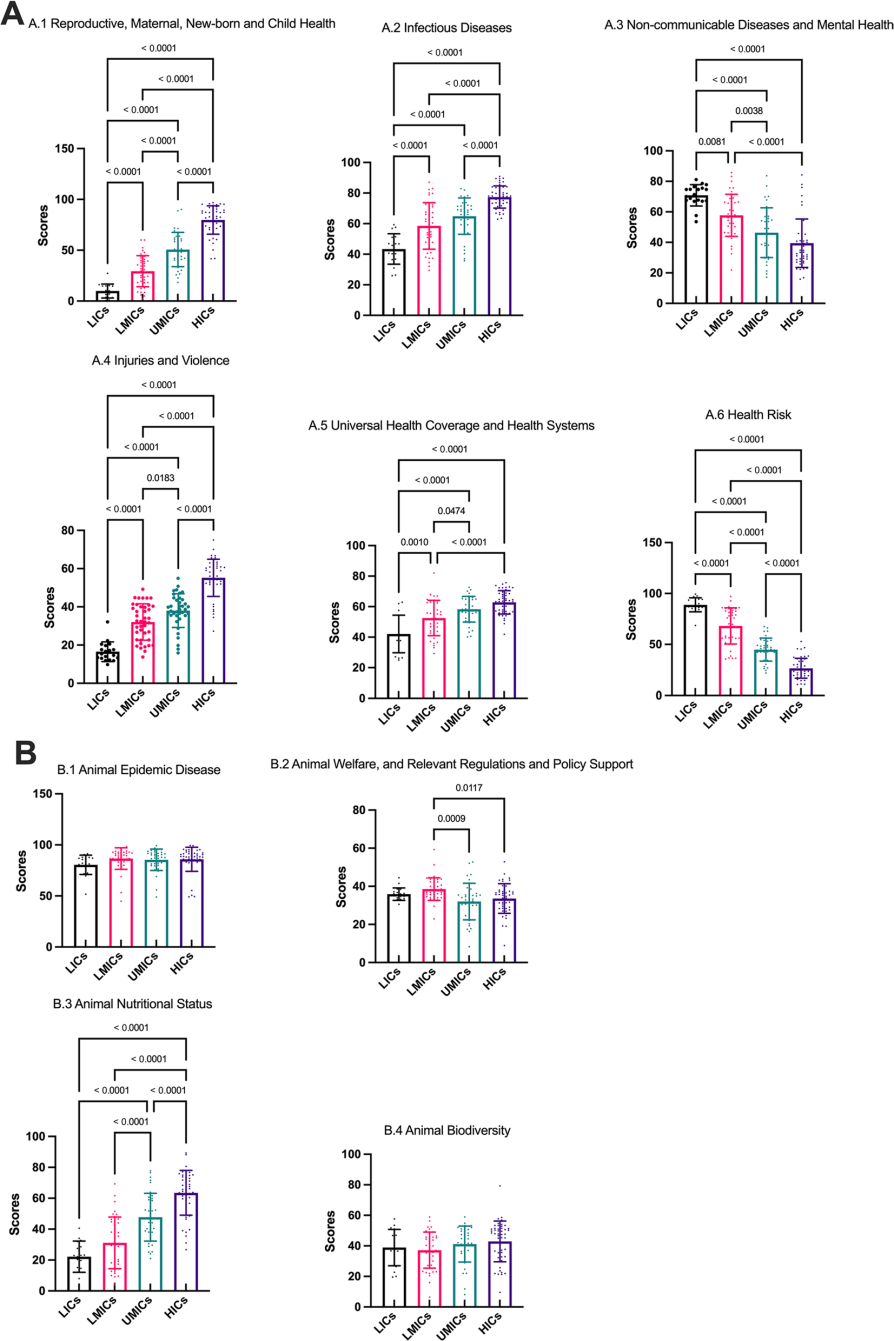

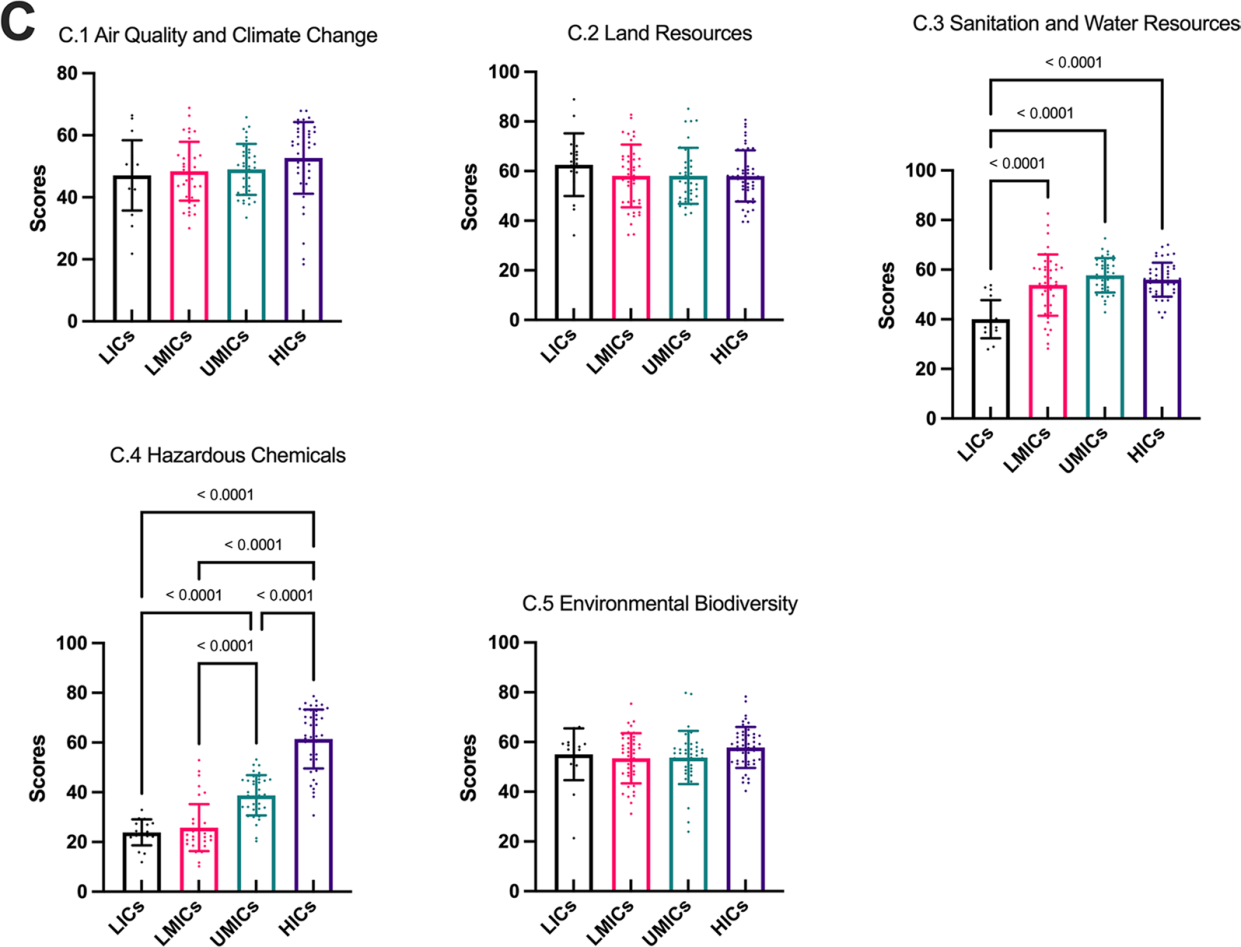
Mean (*SD*) of GOH-IDI and critical indicator scores in different World Bank income groups. The different bar chart colors represent different regions. The error line represents the standard deviation. The dots represent the distribution of the data and correspond to the vertical. Detailed data are listed in Additional file 2. Descriptive analyze are listed in Additional file 3. *SD* standard deviation, *GOH-IDI* Global One Health Intrinsic Driver Index, *LICs* low-income countries, *LMICs* lower middle-income countries, *UMICs* upper middle-income countries, *HICs* high income-countries

The statistical differences between the four World Bank income groups are shown in Fig. 7. Five key indicators are not statistically different under the four World Bank groups, including B.1 Animal Epidemic Disease, B.4 Animal Biodiversity, C.1 Air Quality and Climate Change, C.2 Land Resources and C.5 Environmental Biodiversity. Most of the critical indicators had the highest scores for HICs, while A.3 Non-communicable Diseases and Mental Health and A.6 Health Risk had the lowest scores for HICs and the highest scores for LICs (Fig. 7). Nigeria performs best in Non-communicable Diseases and Mental Health (85.73). Madagascar performs best in Health risk (99.35).

## Discussion

This study analyses the performance of GOH-IDI from multiple organizations from a multidisciplinary perspective to provide the first objective assessment of 146 countries worldwide on the three dimensions of One Health performance. The primary indicators of Human Health, Environmental Health and Animal Health are equally weighted, reflecting the One Health philosophy's emphasis on animal health and ecology. Such a structure also facilitated the identification of problems at a systemic level during this study. This project is based on an authoritative database and optimized by expert consultation and scientific algorithms. This study shows that GOH-IDI is statistically correlated with economic performance (GNI per capita).

HICs countries are more probably to have higher scores in Human Health. LICs countries performed poorly in Reproductive, Maternal, Newborn and Child Health. However, HICs countries performed worse in terms of Health Risk and Non-communicate Disease and Health risk. LICS countries performed worse in Hazardous Chemicals, Reproductive, maternal, newborn and child health and Animal Nutritional Status. These indicators are dependent on national policies and complete industrialization systems. Five indicators are not statistically different between regions at each World Bank income group, including Animal Epidemic Disease, Animal biodiversity, Air quality and Climate Change, Land Resources and Environmental Biodiversity. These indicators may require global policy guidance and concerted efforts to improve climate change and air quality.

Countries in the same World Bank income groups have similar economic and development statuses. Countries with high GOH-IDI scores in each group could function as a model. There are objective differences in GOH-IDI scores between World Bank income groups. However, it is difficult to see the characteristics of these countries from the data only, and an in-depth understanding of their governance systems is needed. Based on the three-level data, the strengths and weaknesses of each country could be analyzed.

(1) Niger has the best GOH-IDI performance in LICs. Niger's great performance may be attributed to its three well-performing second-level indicators, including Non-communicable Diseases and Mental Health, Health Risk, and Land Resources (Fig. 1). Niger focuses on health policies at the national level [24, 25], has a pilot study based on One Health and values the positive effect of systematic research on policy development [26, 27]. The specificity of Niger's performance in GOH-IDI deserves more research analyze and discussion. (2) Uzbekistan has the best GOH-IDI performance in LMICs. Uzbekistan's overall performance in Human Health and Animal Health was great, especially for the three second-level indicators, including Infectious Diseases, Animal Welfare, Relevant Regulations and Policy Support, and Animal Nutritional Status (Fig. 1). Since 1991, Uzbekistan has implemented several major healthcare innovations to improve the efficiency of healthcare services, including healthcare availability, governance, and financing, and to ensure equitable access to healthcare resources [28]. (3) Jordan has the best GOH-IDI performance in UMICs. Jordan's overall performance was more balanced, with five well-performing second-level, including Infectious Diseases, Non-communicable Diseases and Mental Health, Universal Health Coverage and Health Systems, Animal Biodiversity, and Animal Nutritional Status. Jordan has conducted a lot of research and practice on several components of the One Health philosophy, such as antibiotic resistance, human-animal diseases, and climate warming [29–31]. (4) Norway has the best GOH-IDI performance in HICs. Norway performs well in most indicators, with only three that need improvement, including Non-communicable Diseases and Mental Health, Health Risk, and Land Resources (Fig. 1). Norway attaches importance to applying and promoting the One Health concept and economic development [32, 33]. Norway values cross-disciplinary development and introduces the latest technology in practice to aid in the effectiveness of interventions [33].

### Policy recommendations

After analyzing and assessing the global performance of GOH-IDI and its relationship with World Bank income group, the following recommendations might be considered for policymaking in this field.

1. International organizations are needed to take the lead in establishing a global governance paradigm and changing the governance strategies of countries around the globe. Some indicators do not differ significantly between income groups globally (Fig. 7). These indicators are challenging to improve effectiveness through direct economic inputs, and the efforts of individual countries are limited.
2. Increase international and regional cooperation to achieve a win–win situation. Regional differences are apparent, with countries such as Niger, Uzbekistan, Jordan, and Norway being among the top performers in each income group (Fig. 5). The governance paradigms of these countries are valuable for their peers to learn from. In a time of globalization, complementing each other's strengths, strengthening regional multilateral cooperation, and engaging in more international cooperation will be a paradigm for improving the One Health index in the future. As countries and international organizations around the world join One Health-related initiatives and actions, the gaps between countries and regions will narrow and further improve together.
3. Global animal-related databases need increased diversity and data reliability. There are still relatively few animal-related databases (Table 1). Strengthening basic testing facilities and teams is necessary, and reliable data sources combined with professional analyze teams to generate realistic policy recommendations.
4. Strengthening the multilateral consensus and framework for action related to global climate change is necessary. Global climate change is still a big problem. The countries that emit the most greenhouse gases are not necessarily the most affected ones. Climate change has the most significant impact on tropical regions, especially in SSA. Many of the less developed regions in the tropics suffer from extreme climate impacts while needing to fight poverty-induced malnutrition and numerous socioeconomic problems.
5. Combining the One Health concept to develop cross-disciplinary research and practice for efficient and universal policy development. Single discipline and departmental efforts are no longer sufficient to address the current complex international health challenges. The One Health concept guides a multidimensional view of problems. One Health research is directed at three main levels at this stage, including research, implementation, and governance. Area’s worthy of future enhancement include drug resistance traceability, food safety, and climate change. Technical tools worthy of research include early warning systems, One Health strategic policy research, biosafety protection, One Health communication and mass behavior, big data analytics, artificial intelligence, and cloud computing technology applications.

In the process of this study, we noticed that many environmental health and animal health datasets do not have time series and lack the possibility of multidimensional assessment. However, according to our observation, the attention to data is increasing in all world regions. In addition, there are more than 250 countries and regions in the world, and only 146 were included in this study. We will update the existing dataset and integrate a multilingual dataset in a future release.

## Conclusions

The GOH-IDI is an analyze of human health, animal health and environmental health data to assess global performance on each indicator, which helps countries understand their situation, improve global One Health action, and promote balanced development of humans, animals, plants, and ecosystems. The best performing region for GOH-IDI was North America and the worst was sub-Saharan Africa. There is a positive correlation between the GOH-IDI and country economic status, with high-income countries performing well in most indicators. From the perspective of policymakers, they need to find role models with sustainable development strategies that are like their national context to learn from. Case analysis show that some countries have outperformed countries at a similar economic level (GNI per capita) in terms of their GOH-IDI scores. These situations may be related to multiple factors, such as their social and geographical aspects, and require more intensive investigation. For the public health field, this result can facilitate researchers' understanding of the multidimensional situation at a global level and invest more attention in scientific questions that need to be addressed urgently.

## Supplementary Information

**Additional file 1**: Full scheme for the indicators and weights of GOH-IDI.

**Additional file 2**: GOH-IDI complete data and regional classification.

**Additional file 3**: Descriptive statistics summary.

## Data Availability

The full study protocol and the datasets are available, following manuscript publication, upon request from the corresponding author (Shi-Zhu Li, lisz@chinacdc.cn).

## Abbreviations

COVID-19: Coronavirus disease 2019
EAP: East Asia and Pacific
ECA: Europe and Central Asia
EPI: Environmental Performance Index
FAO: Food and Agriculture Organization
FAHP: Fuzzy Analytical Hierarchy Process
GOHI: Global One Health Index
GOH-IDI: Global One Health Intrinsic Driver Index
GBD: Global Burden of Disease
HIC: High income-country
IHME: Institute for Health Metrics and Evaluation
LAC: Latin America and the Caribbean
LIC: Low-income country
LMIC: Lower middle-income country
MENA: Middle East and North Africa
NA: North America
OIE: World Organization for Animal Health
SA: South Asia
SDGs: Sustainable Development Goals
SSA: Sub-Saharan Africa
UMIC: Upper middle-income country.
WHO: World Health Organization
